# A human case of spotted fever caused by *Rickettsia parkeri* strain Atlantic rainforest and its association to the tick *Amblyomma ovale*

**DOI:** 10.1186/s13071-019-3730-2

**Published:** 2019-10-11

**Authors:** Anaiá da Paixão Sevá, Thiago Fernandes Martins, Sebastián Muñoz-Leal, Ana Carla Rodrigues, Adriano Pinter, Hermes R. Luz, Rodrigo N. Angerami, Marcelo B. Labruna

**Affiliations:** 1 Universidade Estadual de Santa Cruz, Ilhéus, BA Brazil; 2 Departamento de Medicina Veterinária Preventiva e Saúde Animal, Faculdade de Medicina Veterinária e Zootecnia, Universidade de São Paulo, São Paulo, SP Brazil; 3 Universidade do Estado de Mato Grosso, Campus Nova Xavantina, Nova Xavantina, MT Brazil; 4 Superintendência de Controle de Endemias do Estado de São Paulo, São Paulo, SP Brazil; 5 Departamento de Patologia, RENORBIO, Universidade Federal do Maranhão, São Luís, MA Brazil; 6 Hospital das Clínicas, Universidade Estadual de Campinas, Campinas, SP Brazil

**Keywords:** *Rickettsia parkeri*, *Amblyomma ovale*, Inoculation eschar, Bahia

## Abstract

**Background:**

*Rickettsia parkeri* strain Atlantic rainforest has emerged in Brazil during the last 10 years, with three laboratory-confirmed human cases. While these cases were epidemiologically associated with the tick *Amblyomma ovale*, in none of them the tick specimens that bit the patients could be identified.

**Results:**

We report a clinical case of spotted fever rickettsiosis that was acquired in an Atlantic forest area in Bahia state, northeast Brazil. The case was determined to be caused by *R. parkeri* strain Atlantic rainforest, based on molecular analysis of the crust removed from the tick bite site (inoculation eschar) of the patients’ skin. DNA extracted from the crust yielded partial sequences of three rickettsial genes (*gltA*, *ompA* and *ompB*), which were 99–100% identical to *R. parkeri* strain Atlantic rainforest. The tick specimen that was attached to patient skin was identified as a female of *A. ovale.*

**Conclusions:**

We report the fourth confirmed case of spotted fever rickettsiosis caused by *R. parkeri* strain Atlantic rainforest, providing to our knowledge for the first time, direct evidence of *R. parkeri* strain Atlantic rainforest transmission by *A. ovale*.

## Background

Tick-borne rickettsioses are zoonoses caused by bacteria of the genus *Rickettsia*, especially species belonging to the spotted fever group (SFG) [1]. While some SFG agents have been reported since the first half of the last century, an increasing number of other rickettsiae have been reported only recently, as etiological agents of emerging diseases in different parts of the world [1]. Generally, SFG diseases are clinically characterized by acute fever, skin lesions such as rash and/or an inoculation eschar, and non-specific signs [2].

A new SFG agent, *Rickettsia parkeri* strain Atlantic rainforest, has emerged in Brazil during the last ten years, with three laboratory-confirmed clinically mild cases, one in the São Paulo State [3], one in Bahia State [4] and one in Santa Catarina State [5]. While the identity of the agent of these three cases was initially reported as an unnamed *Rickettsia* species (*Rickettsia* sp.) with different strain names (Atlantic rainforest or Bahia), a recent phylogenetic study concluded that it corresponds to a single species and strain, named as *R. parkeri* strain Atlantic rainforest [6].

A laboratory study demonstrated that the tick *Amblyomma ovale* is a competent vector of *R. parkeri* strain Atlantic rainforest [7]. In fact, the three confirmed cases of the disease in Brazil were epidemiologically associated with *A. ovale*, based on the findings of *R. parkeri* strain Atlantic rainforest-infected *A. ovale* ticks in the environment or/and infesting domestic dogs from the same areas where the patients reported to have acquired the infected ticks [8–10]. However, the tick specimens that bit the patients could not be identified in any of the three laboratory-confirmed cases.

Here, we report the fourth confirmed case of SFG rickettsiosis caused by *R. parkeri* strain Atlantic rainforest and, to our knowledge, provide for the first time a direct association with the bite of an *A. ovale* tick.

## Methods

### Case presentation

**Fig. 1:**
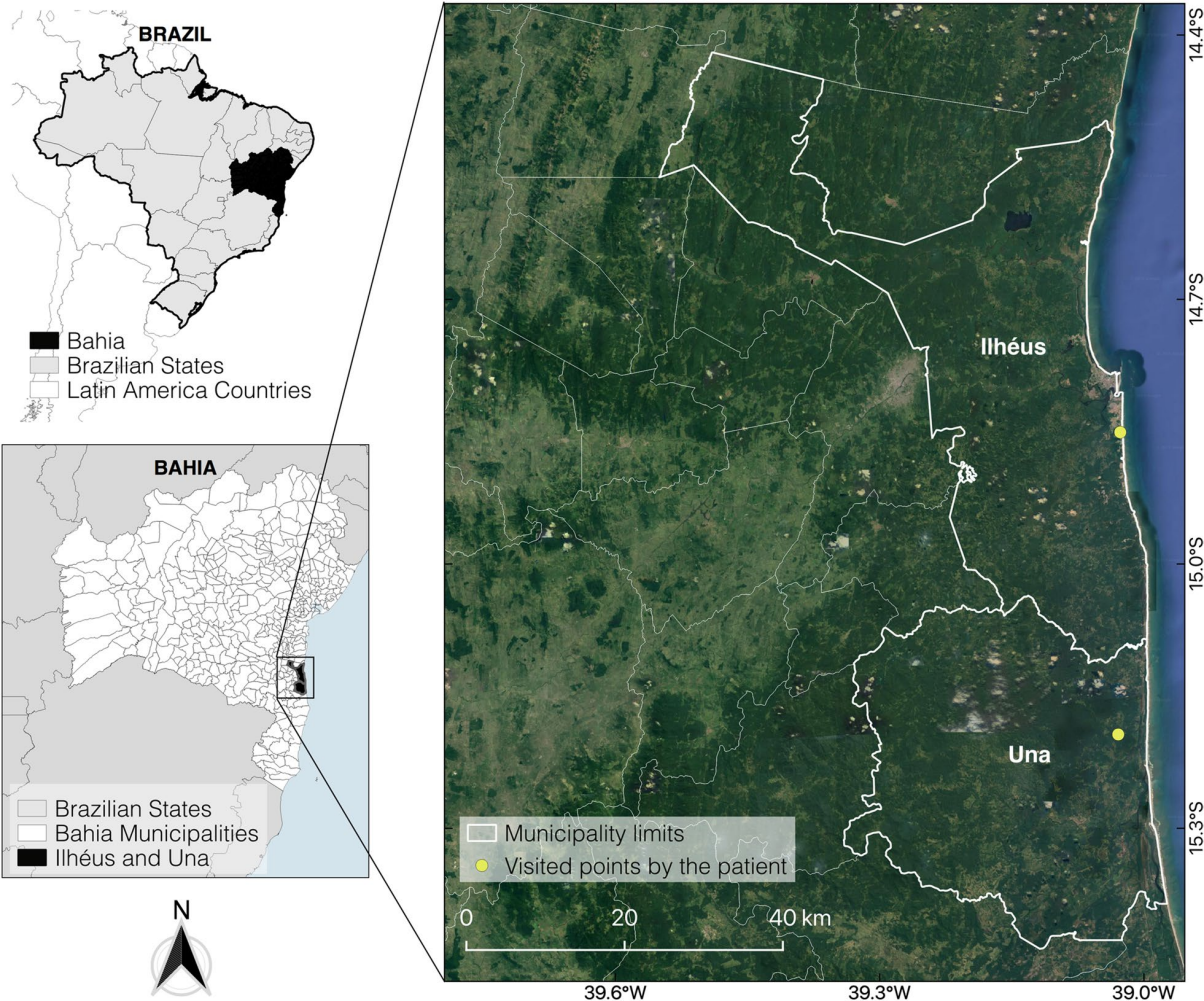
Visited points (Ilhéus and Una) by the patient at the day of the tick bite

**Fig. 2:**
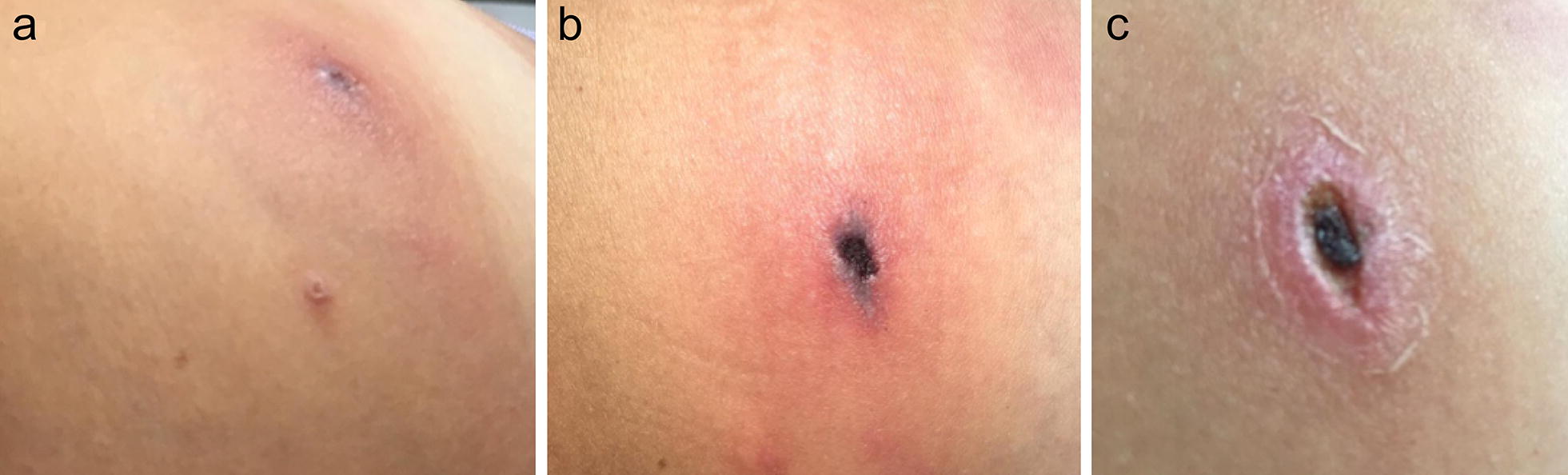
Inoculation eschar at the tick bite site on the left iliac region of a patient infected with *Rickettsia parkeri* strain Atlantic rainforest in Bahia State, northeastern Brazil. **a** 8 days after the tick bite (DATB), **b** 11 DATB, **c** 19 DATB

A 31-year-old Brazilian white woman was bitten by a tick on her left iliac region on December 6, 2018, while visiting two areas of Ilhéus and Una municipalities of the Atlantic rainforest biome in the south of Bahia State, northeast Brazil (Fig. 1). She only noticed the tick attached to her lower iliac region at the night of that same day, and thought it might have been attached to her skin for about 12 hours. The attached tick was photographed, detached and discarded. On December 12 (day 6 after the tick bite) she presented acute clinical signs and symptoms including a papular lesion (12 × 7 mm) surrounded by a macular rash with a necrotic central lesion and deep pain at the tick bite site (inoculation eschar) (Fig. 2), intense arthralgia mainly in the left leg, regional lymphadenopathy (inguinal), myalgia, malaise, nausea, diarrhea, constant headache and the feeling of fever, not confirmed by body temperature measurement.

On day 6 of symptoms (December 17), the patient was examined by a physician who prescribed cephalexin (500 mg, PO, q6hr) and analgesic every four hours, both for seven days. Results of the hemogram and blood biochemistry at December 17 were unremarkable, except for discrete leukopenia [4400 /mm^3^ (reference values: 5000–10,000/mm^3^)] and low number of eosinophils [44/mm^3^ (reference values: 100–400/mm^3^)]. On the next day (day 7 of symptoms, December 18) most symptoms excepting arthralgia resolved. The arthralgia ceased only at December 29. The eschar was completely healed 40 days after the tick bite.

## Results

Since the patient was already in contact with the technical staff of our laboratory for other purposes before she became ill, she was informed by some of us that her illness could be spotted fever. Therefore, on January 03, 2019 (22 days after symptom onset) she self-collected, manually pulling the crust of the inoculation eschar, stored it in a sterile microtube with 96% ethanol, and sent to our laboratory for molecular analysis. DNA of this crust was extracted using a DNAeasy Blood and Tissue Kit (Qiagen, Valencia, CA, USA), and was tested by different protocols of polymerase chain reaction (PCR) targeting three rickettsial genes as follows: primers CS-78 and CS-323, and CS-239 and CS-1069, targeting two overlapping fragments (401 bp and 830 bp) of the rickettsial *gltA* gene [11]; primers Rr190.70F and Rr190.701R, targeting a 632-bp fragment of the rickettsial *ompA* gene [12]; and primers 120-M59 and 120-807, targeting a 862-bp fragment of the rickettsial *ompB* gene [13]. Amplicons of the expected size were generated by the all PCR assays. PCR products were treated with ExoSAP-IT (USB, Cleveland, OH, USA) and sequenced in an ABI automated sequencer (ABI Prism 3500 Genetic; Applied Biosystems, Foster City, CA, USA). After BLAST analyses (http://blast.ncbi.nlm.nih.gov/Blast.cgi), the resultant sequences of *gltA* (1081-bp) and *ompB* (817-bp) were shown to be 100% identical to GenBank sequences of *R. parkeri* strain Atlantic rainforest (GQ855235 for *gltA*, KU882101 for *ompB*), whereas the *ompA* sequence was 99.8% (589/590-bp) identical to *R. parkeri* strain Atlantic rainforest (MF536975). The *gltA*, *ompA* and *ompB* sequences generated in this study were deposited in GenBank under the accession numbers MN027564, MN027565 and MN027566, respectively.

Paired blood samples for serologic analysis were collected from cephalic vein on day 19 and day 41 (December 30, 2018 and January 21, 2019, respectively) from disease onset. The serum samples were tested by immunofluorescence assay (IFA) against crude antigens of four *Rickettsia* species from Brazil [*R. parkeri* strain Atlantic rainforest, *R. parkeri* (*sensu stricto*) strain At24, *Rickettsia amblyommatis* strain Ac37, *Rickettsia rickettsii* strain Taiaçu], as previously described [8, 9]. Briefly, sera were diluted in 2-fold increments with phosphate-buffered saline, starting from the 1:64 dilution. Slides were incubated with fluorescein isothiocyanate-labeled goat antihuman IgG (Sigma, St. Louis, MO, USA). For the two serum samples, the end point IgG antibody titer reacting with each of the four *Rickettsia* antigens was determined. Both sera displayed highest endpoint titers to *R. parkeri* strain Atlantic rainforest and *R. parkeri* (*sensu stricto*) strain At24 (Table 1).

**Table 1:** *Rickettsia* spp. serologic endpoint titers by immunofluorescence assay (IFA) for a Brazilian patient that was bitten by a tick on December 6th, 2018 in Bahia state, Brazil

Antigen	Titer	
	December 30th, 2018	January 21st, 2019
*R. parkeri* Atlantic rainforest	1024	1024
*R. parkeri* (*sensu stricto*) At24	512	1024
*R. rickettsii*	256	256
*R. amblyommatis*	256	128

When the patient noticed the attached tick, she photographed it by using her cell phone. The tick was immediately removed, and unfortunately discarded. Even though the picture had a bad resolution (data not shown), we were able to identify the attached tick as a female of *A. ovale* based on its elongate body and the typical scutal ornamentation pattern, characterized by a strong light yellowish mark at the posterior border of the scutum. It is interesting to note that a second *A. ovale* female was found attached to the nape of one of our colleagues during January 2019, while he was walking in the Atlantic rainforest of Ilhéus. The tick remained attached for only a few minutes, our colleague did not become ill, and he sent us the tick specimen, which was confirmed as *A. ovale* and was deposited in our tick collection “Coleção Nacional de Carrapatos Danilo Gonçalves Saraiva”, São Paulo, Brazil, under accession number CNC-3929.

## Discussion

We confirm by molecular analyses the fourth clinical case of spotted fever caused by *R. parkeri* strain Atlantic rainforest in Brazil. The clinical pattern of the current case was also reported in the previous three cases [3–5], except for the diarrhea, which is reported here for the first time. The observed incubation period of six days is shorter than the seven to ten days reported for the previous three cases; however, it is within the incubation period of several other SFG rickettsioses, ranging from four to ten days [1]. Our serological analyses revealed similar endpoint titers to all tested *Rickettsia* species, albeit with highest titers to *R. parkeri.* This lack of increase of the endpoint titers between the two serum samples could be understood and indeed was related to the time points of serum collection, both at the convalescent phase with a late collected first sample, thus precluding a demonstration of seroconversion.

To our knowledge, we demonstrated for the first time that parasitism by an *A. ovale* tick was clinically associated to a mild spotted fever case, since the inoculation eschar developed exactly at the same site where the tick was attached six days prior to the onset of clinical signs. This finding supports previous reports that provided epidemiological evidence of *A. ovale* as the main vector of *R. parkeri* strain Atlantic rainforest to humans [8–10]. In addition, a recent study in the rural area of Ilhéus (same area of the present study) reported that 15% of the *A. ovale* ticks collected from domestic dogs were infected by *R. parkeri* strain Atlantic rainforest, with serological evidence that some of these dogs had been infected by *R. parkeri* [14]. In conjunction, these results support the occurrence of a potentially endemic natural focus of *R. parkeri* strain Atlantic rainforest in the area of the present study, highlighting the potential of unknown previous human cases and imminent risk of further human cases of spotted fever in this area.

In the present case, the patient initiated a therapy with cephalexin on the sixth day of illness, and reported a significant improvement from the next day. At a first sight, this information might indicate a good efficacy of cephalexin to treat this spotted fever case. However, it has been widely reported that cephalosporins (including cephalexin) are inactive against rickettsial agents [15]. Thus, we believe that the general improvement of the patient was due, in fact, to the known mild virulence of this incriminated rickettsial species, and in some degree to host’s immunological response against *R. parkeri*, an agent related to an acute, mild and self-limiting disease that has not resulted in any fatalities or more complicated clinical outcomes so far, even without specific antimicrobial treatment [1]. Nevertheless, it is noteworthy that the physician of the present case did not suspect tick-borne rickettsiosis, even though the patient was presented with an inoculation eschar at the site of the tick-bite. Indeed, lack of suspicion of rickettsiosis has been usually associated to the fatal outcome of more severe tick-borne rickettsiosis, such as Brazilian spotted fever, caused by *Rickettsia rickettsii* [16].

The tick *A. ovale* has a wide distribution in Latin America, from Mexico to Argentina [17]. Within this distribution, there have been multiple reports of *R. parkeri* strain Atlantic rainforest-infected *A. ovale* ticks in countries besides Brazil, such as Argentina [18], Colombia [19], Belize [20] and Nicaragua [21]. In the latter country, *R. parkeri* strain Atlantic rainforest was equivocally reported as *R. africae*, but the correct identity of the agent can be verified by its sequences that were deposited in GenBank, 100% identical to the former agent. Given the widespread distributions of both *A. ovale* and *R. parkeri* strain Atlantic rainforest, coupled with the highly anthropophilic habits of the adult stage of *A. ovale* [22], it is believed that the spotted fever caused by this agent has remained underreported in known transmission localities and unreported in many areas. The case here reported is a classic example. Had the patient not contacted our research group, referral for rickettsial agents, at the time of the acute febrile illness, the case would have remained as one more acute febrile syndrome without an etiological diagnosis. The need of a structured epidemiological surveillance and laboratory resources able to detect and to investigate suspected cases should be highlighted, for a better comprehension of the real epidemiology of the *R. parkeri* strain Atlantic rainforest spotted fever as an emerging tick-borne disease in Latin America.

## Conclusions

We report the fourth confirmed case of spotted fever rickettsiosis caused by *R. parkeri* strain Atlantic rainforest. To our knowledge, we provide for the first time, direct evidence of *R. parkeri* strain Atlantic rainforest transmission by *A. ovale*.

## Data Availability

The data generated during this study are included within this manuscript or are available upon request from the corresponding author. The newly generated sequences were deposited in the GenBank database under the accession numbers MN027564 (*gltA*), MN027565 (*ompA*) and MN027566 (*ompB*).

## Abbreviations

SFG: spotted fever group
PCR: polymerase chain reaction
IFA: immunofluorescence assay
